# Friend of a Friend with Benefits ontology (FOAF+): extending a social network ontology for public health

**DOI:** 10.1186/s12911-020-01287-8

**Published:** 2020-12-15

**Authors:** Muhammad Amith, Kayo Fujimoto, Rebecca Mauldin, Cui Tao

**Affiliations:** 1 School of Biomedical Informatics, The University of Texas Health Science Center at Houston, 7000 Fannin St Suite 600, Houston, TX 77030 USA; 2 School of Public Health, The University of Texas Health Science Center at Houston, 7000 Fannin Street, Suite 2514, Houston, TX 77030 USA; 3 The University of Texas at Arlington, 211 South Cooper Street, Box 19129, Arlington, TX 76019 USA

**Keywords:** Ontology, Public health, Disease surveillance, Social network analysis, HIV

## Abstract

**Background:**

Dyadic-based social networks analyses have been effective in a variety of behavioral- and health-related research areas. We introduce an ontology-driven approach towards social network analysis through encoding social data and inferring new information from the data.

**Methods:**

The Friend of a Friend (FOAF) ontology is a lightweight social network ontology. We enriched FOAF by deriving social interaction data and relationships from social data to extend its domain scope.

**Results:**

Our effort produced Friend of a Friend with Benefits (FOAF+) ontology that aims to support the spectrum of human interaction. A preliminary semiotic evaluation revealed a semantically rich and comprehensive knowledge base to represent complex social network relationships. With Semantic Web Rules Language, we demonstrated FOAF+ potential to infer social network ties between individual data.

**Conclusion:**

Using logical rules, we defined interpersonal dyadic social connections, which can create inferred linked dyadic social representations of individuals, represent complex behavioral information, help machines interpret some of the concepts and relationships involving human interaction, query network data, and contribute methods for analytical and disease surveillance.

## Background

Social network analysis is defined as a “broad strategy for investigating social structures” [1] and a “set of techniques used to understand these relationships and how they affect behaviors” [2]. Qualitative analyses and measures can help us interpret these network structures and understand the underlying behaviors and influences among individuals [3]. Furthermore, other methods like actor-oriented network dynamic modeling methodology can further analyze social network structures [4]. Social network analysis has long had an impact on such public health research areas as social support, HIV/STIs, family planning and reproductive health, community health, and inter-organizational relations [2].

Ontologies and social networks share some features. Both express information in graph-like representations, yet each has its own way to elicit information. Ontologies are representational artifacts of domain knowledge in an electronic format. It abstract complex information pertaining to concepts, data, entities, properties, etc., and it use logical links between them that can imbue meaning to data and knowledge. In addition, because ontologies provide formal, normalized and semantic definitions, data can be standardized allowing for sharing and consistency/interoperability between researchers. As an electronic artifact, the knowledge is machine-understandable, which allows machines to perform reasoning, such as inferring new relationships and conducting automatic classification. Social network graphs, from a social network analysis perspective, represent individuals as nodes, with connecting edges that expresses some behavioral or social structure among the individuals. Aside from qualitative observation of these networks, statistical methods or metrics (e.g. centrality, clustering, etc.) can highlight some important knowledge about the social network of the individuals.

While the bulk of ontology research is dedicated to the life sciences [5], ontologies have not enjoyed the same widespread application into the public health domain as social network analysis, yet it has the potential to make important contributions to public health research and practice [6]. Ontologies can describe very complex information beyond direct links between two nodes. In addition, we can link an ontology with data to help describe the complex relationship within data. If we link an ontology to social network survey data, we could infer new dyadic connections between individuals using the ontology’s reasoning capabilities and defined decision rules.

**Fig. 1:**
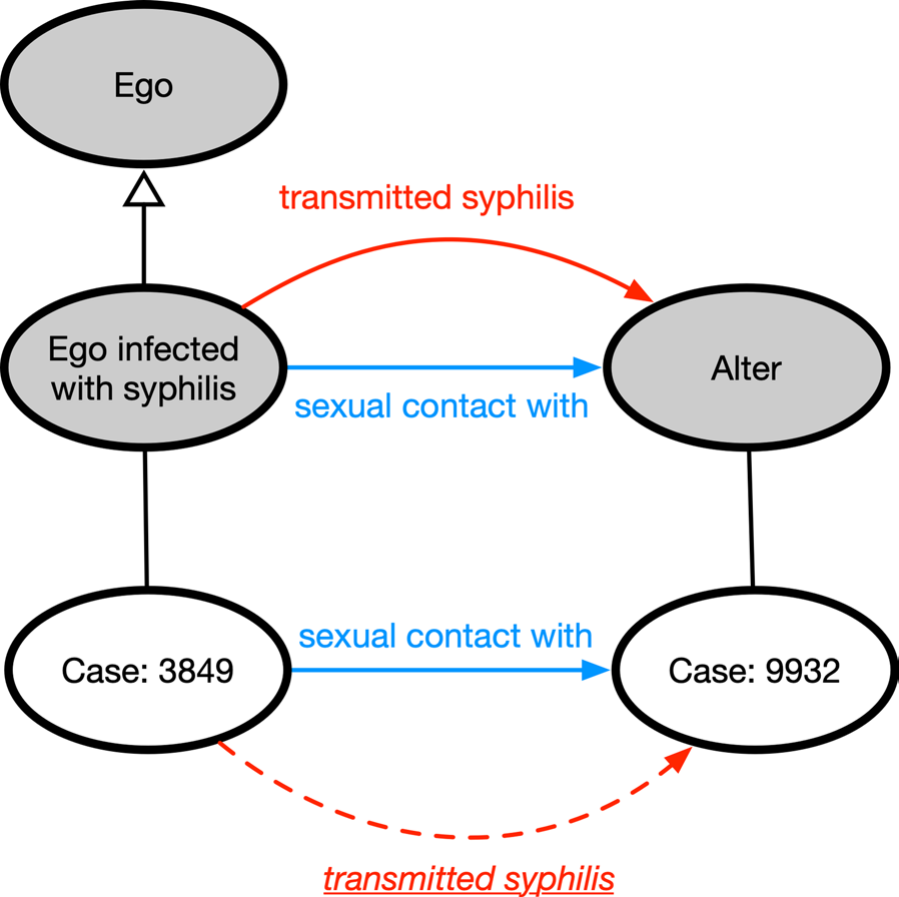
Linking survey data with ontology. Survey participants and their alters are identified by case identifier

For example, in Fig. 1, we are presented with two hypothetical classes for *ego* (Ego infected with syphilis) and *alter* (Alter). An *ego*, in social network analysis, indicates a focal individual in a network, and an *alter* is an individual with whom the ego has a relationship or social tie. Between the classes, there is sexual contact with and “transmitted syphilis” relationship defined on the TBox level. If we instantiate or link these two classes with participant survey data of social network of sexual interaction, and explicitly define individuals having sexual contact with one having syphilis (as shown in Fig. 1), we can assume with some chance that there is a transmission of syphilis between the individuals. Figure 1 shows a inferred relationship of “transmitted syphilis”.

**Fig. 2:**
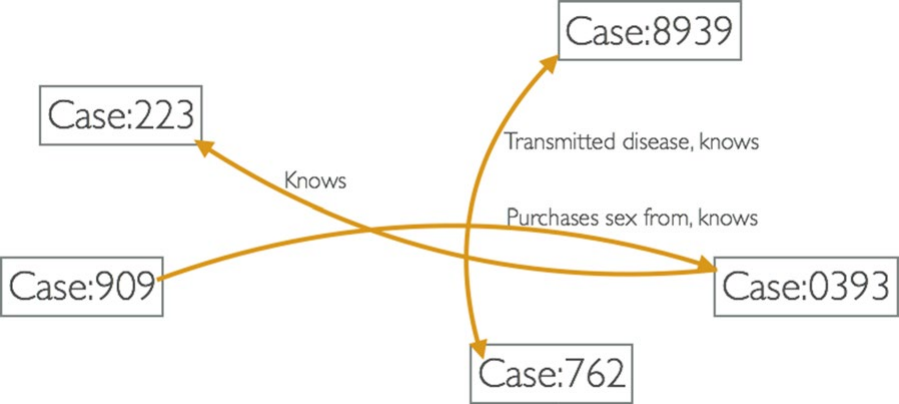
Expressed inferred social network from information

Thus, with the support of an ontology and with an extensive survey of many participants, we would have the potential to generate a whole network of comprehensive connections among people from the survey data, ranging from types of “knowing” relationships to disease transmission and sex work (Fig. 2). This paper will focus on a method to achieve this.

This paper presents an application ontology for social network analysis that was constructed to describe the domain of social and sexual behavior as it pertains to STI transmission between individuals. It also proposes future implementations to annotate social network data to discover new knowledge and new links of information on a particular level. By modeling this information in a formal ontology, we may be able to reveal new links and assumptions based on the data. In addition, this work could provide standardization of this domain to assist in future research to further the use of ontologies in social network analysis for public health.

### Related studies

We reviewed studies from 2012 to the present that used ontologies for social network analysis, specifically for public health research. We searched PubMed, ACM Library, and IEEE Xplorer databases with the query “social network analysis” AND (ontology OR “semantic web”). The search yielded three papers from PubMed, 17 from ACM Library, and six from IEEE Xplorer. Results with no relevance to the health domain or no relevant mention of ontologies and social network analysis (i.e., development or utilization of ontologies for analysis of social structures) were excluded. Papers that at least discuss ontologies for social network analysis were included. Four papers met the criteria.

One paper discussed the utility and review of semantic web and ontologies for social network analysis with some discussion of the usefulness of SPARQL queries to generate centrality measures [7]. Vacca and colleagues utilized the VIVO ontology to produce a dataset of researchers from the University of Florida, and they subsequently proposed the use of an alteration network intervention to produce network links for creative collaboration [8]. Rajabi and Abidi utilized a surgery discussion board to create a one-node from a two-node network of surgeon users and discussion topics [9]. They used semantic similarity measures to determine the influential discussion users, on par with traditional social network analysis techniques, from the one-node network.

From the four papers reviewed, it was apparent that most used ontologies as a supporting artifact, either as a dataset or as a component that augmented a system [8–10]. While three of the four had some impact, only one showed real impact in public health [10]. None of the papers utilized any machine reasoning features offered by the ontology or performed an approach similar to what we propose.

## Methods

**Fig. 3:**
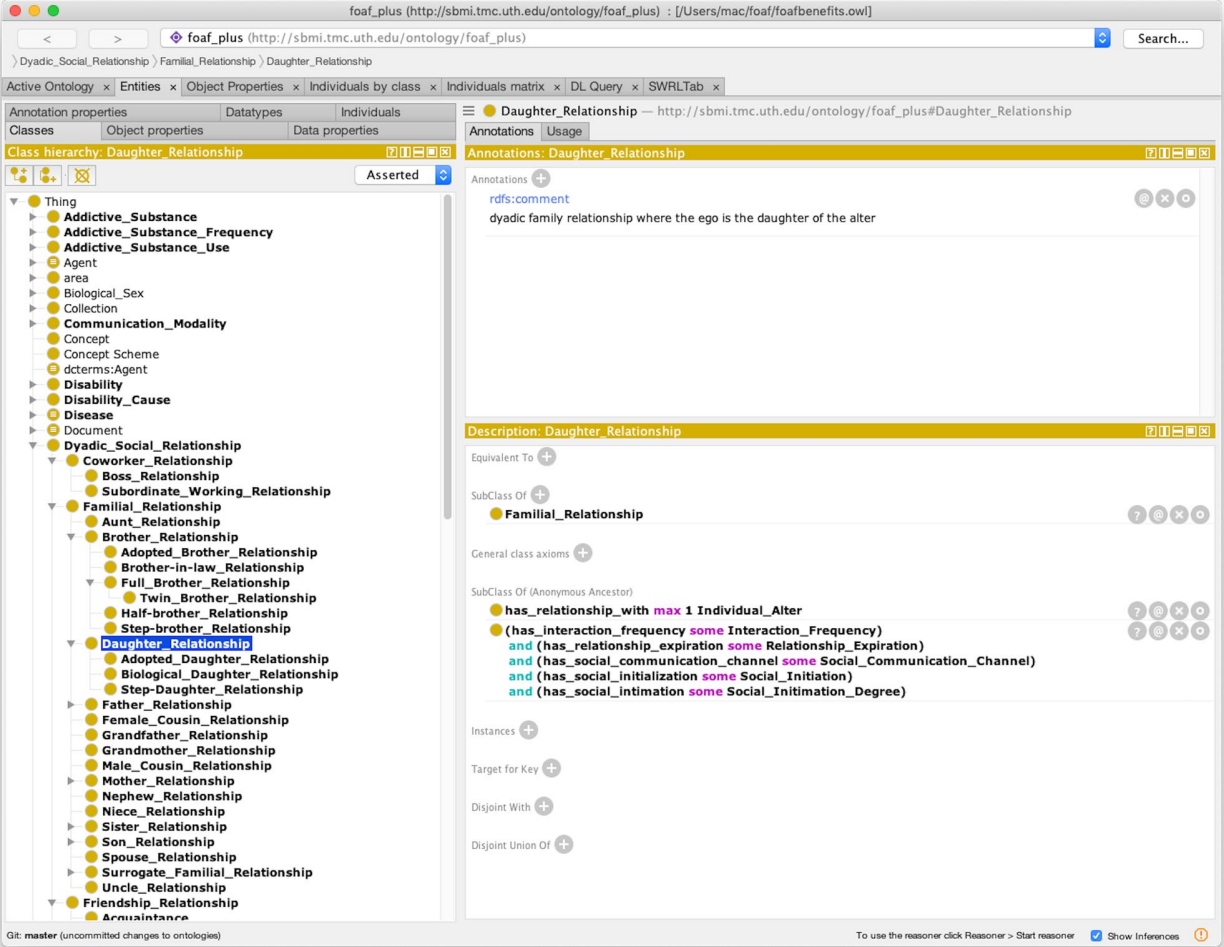
Protégé editor with FOAF+ loaded

Our Friend of a Friend with Benefits Ontology (FOAF+), is designed to address the lack of a social network-related ontologies for use in the field of public health. Currently, there is a lightweight social ontology called Friend of a Friend (FOAF) [11] that describes basic friend-based relations between people. It involves linking information to represent social networks, representational networks, and information networks [12]. To create an ontology with specific health-related application, we added health-related concepts to the FOAF ontology. Enriching FOAF in this way uses the best practice of building on an existing model and incorporating other ontologies to foster sharing, interoperability, and re-usability [13]. FOAF+ describes types of social ties and interactions not included in FOAF. In addition to adding new types of social ties and interactions to FOAF, we ascribe to each entity new features or attributes. These attributes were extracted from the ADD (Adolescent to Adult) Health project survey forms [14] and from YMAP (Young Men’s Affiliation Project of HIV Risk and Prevention Venue) [15], a project that examines the social networks of minority Young Men Who Have Sex with Men (YMSM). These two projects are large-scale public health research projects that include a wide range of health-related variables in order to understand disease transmission and social behavior. We used Stanford’s Protégé [16] to encode the ontology (Fig. 3) and its built-in reasoner feature to display the inferred relationship and information (see “Results” section), and conferred with our public health co-authors on the design of concepts.

We also imported the Food and Agriculture Organization’s geopolitical ontology [17] for a list of countries; however, within the confines of this study it was barely needed. We also imported the Ontology of General Medicine Sciences (OGMS) and Ontology of Medically Related Social Entities (OMRSE) [18] to allow for linking of similar concepts, specifically for the personal medical information. For the time being, our initial priority was to design an application ontology to produce inferred networks from social data. Therefore, aligning to existing standards and ontologies will be a later concern. Examination of the entire ontology can be found through this link, which provides an additional minimal version (foafbenefits-minimal) and full version with imports (foafbenefits-full), along with the basic variant: http://bit.ly/foafplus.

**Fig. 4:**
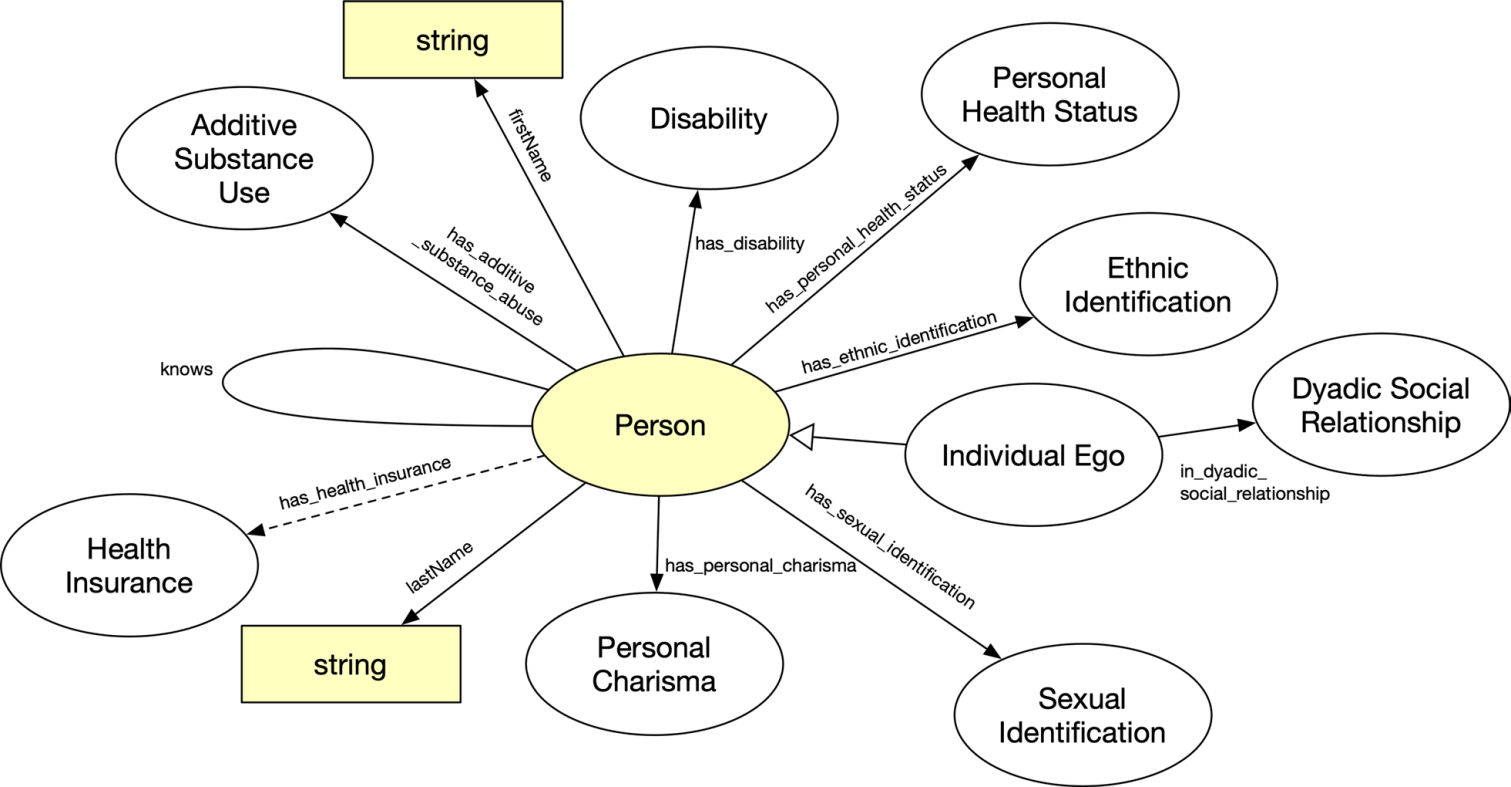
Person class where classes are represented as circles and rectangles as data value types for data properties. Yellow highlights represents original FOAF ontology concept and property

In FOAF+, most of the concepts from the FOAF remain. As mentioned, we enriched FOAF with additional concepts and links. For example, the Person concept, central to the ontology, is linked to health-related concepts such as disability, health status, and substance use as shown in Fig. 4.

**Fig. 5:**
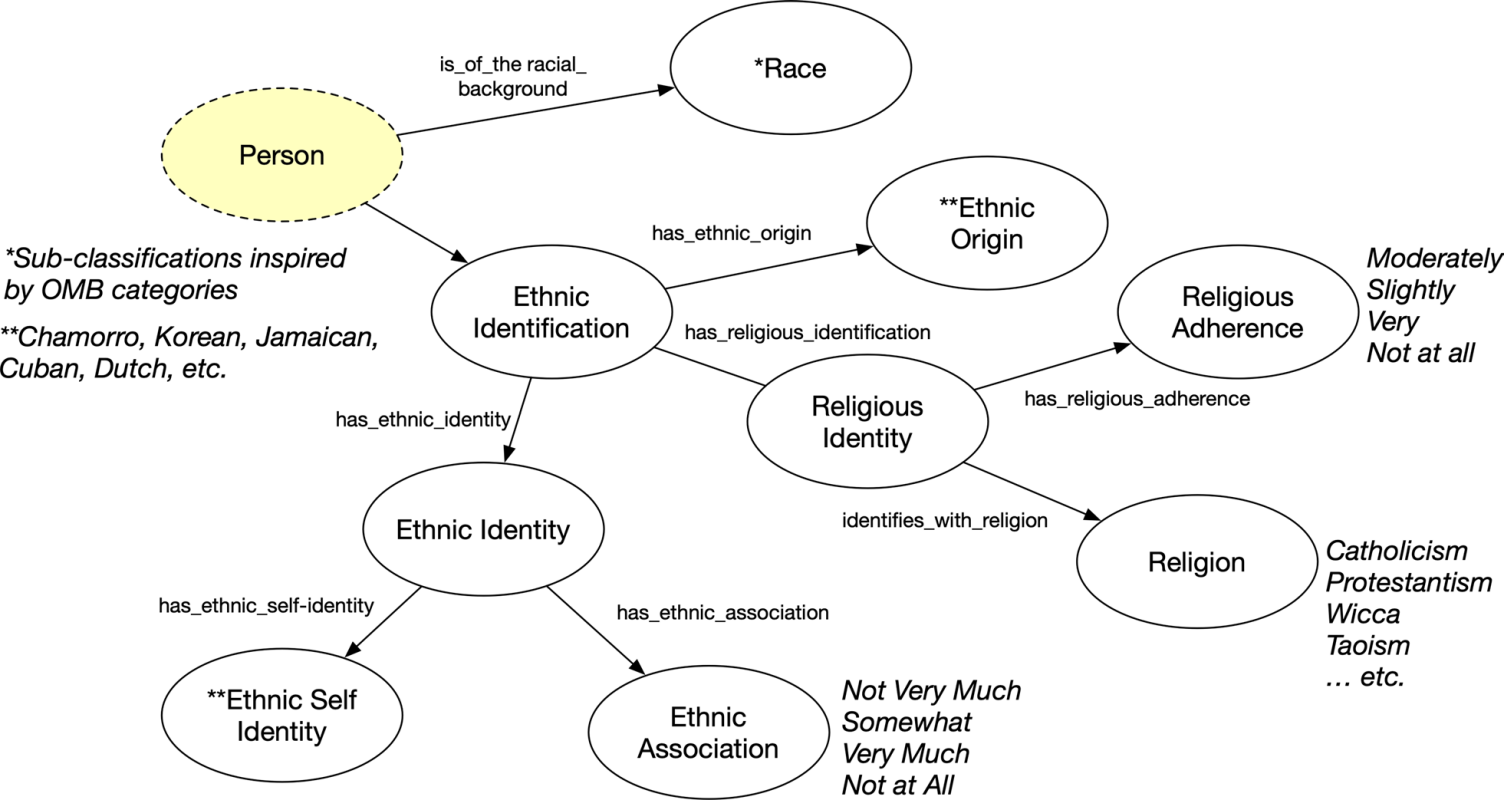
Race and Ethnic Identification concepts where classes are represented as circles and rectangles as data value types for data properties. Yellow highlights represents original FOAF ontology concept and property

### Race, ethnicity, and sexual identity

Apart from attribute-style graphs, we used n-ary patterns extensively [19] to describe the various social-behavioral information and knowledge of the survey participants. To exemplify, Ethnic Identification involved various definitions and descriptions of which ethnicity individuals identified (Fig. 5). Any identification involving birth ethnicity, how the person identified, and the degree to which he/she associated with the ethnicity (Ethnic Association). The same is seen with Religious Identity. For example, the faith a person practices (Religion) and the extent to which he/she adheres to the faith (Religious Adherence). We also include Race to distinguish from the more granular ethnic classification. A survey participant may be of the racial category of black, and have an ethnic identity and origin pertaining to being Cuban (i.e. “Afro Cubans”).

**Fig. 6:**
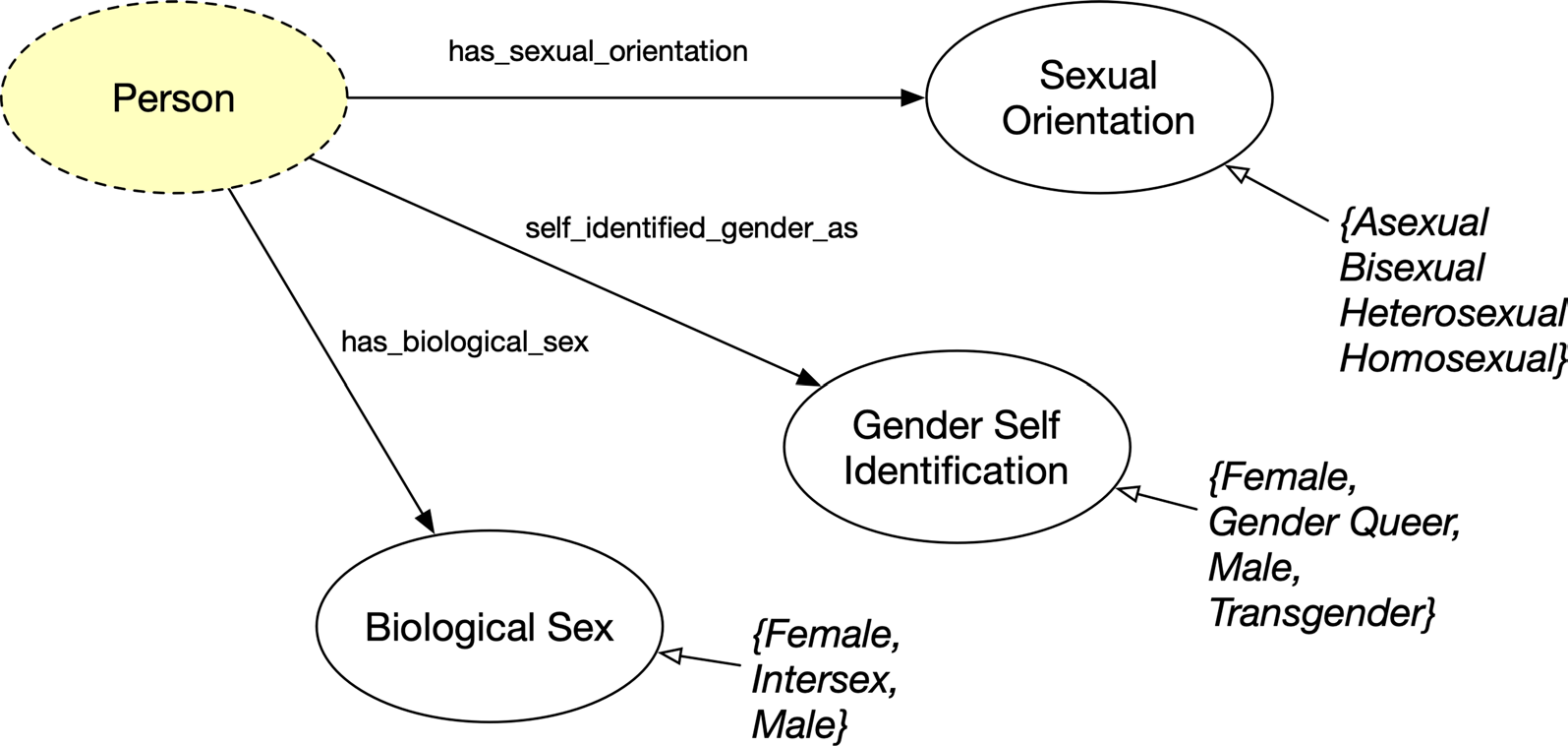
Gender and sexual identification concepts where classes are represented as circles and rectangles as data value types for data properties. Yellow highlights represents original FOAF ontology concept and property

In FOAF+, a Person may have a sexual orientation describing their personal attraction (Sexual Orientation), biological sex (Biological Sex), and the gender to which they identify (Gender Self Identification). Figure 6 shows these three concepts related to sexual identification.

**Fig. 7:**
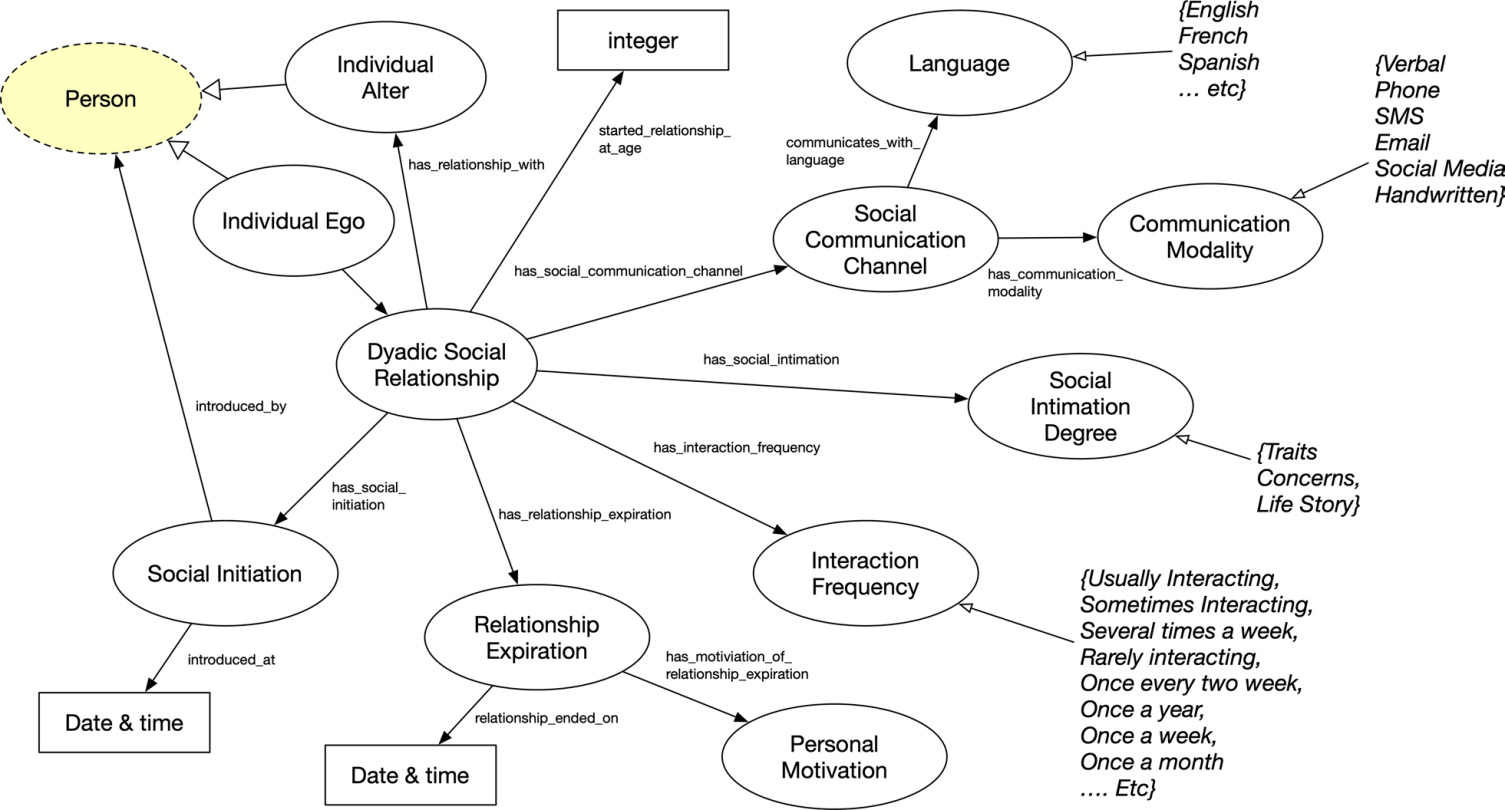
Dyadic Social Relationship concept where classes are represented as circles and rectangles as data value types for data properties. Yellow highlights represents original FOAF ontology concept and property

### Dyadic social and sexual relationships

Dyadic Social Relationship was another important concept for FOAF+ (Fig. 7). Based on survey prompts and data, this concept elaborates relationship details between individuals of those surveys. A person, specifically the *ego* in social network terminology, has a social relationship, which points to (“has relationship with”) another party, the *alter* in the relationship. In McAdams’s 1996 study [20], two individuals who know each other (Social Intimation Degree) in a social relationship can be measured on three levels: Traits (knowing the person’s features), Concerns (knowing the person’s tasks, motivations, beliefs), and Life Story (knowing the person’s historic evolution). Along with the social, interactions involve a means of communication (Social Communication Channel), the language communicated (Language), and the mode (Communication Modality). The interactions also include how frequently two individuals interact (Interaction Frequency) and how they first became acquainted (Social Initiation), who introduced them and how much time they spend together. Relationship Expiration, on the other hand, describes why and when the relationship ended (Personal Motivation). This design pattern includes some data properties for date and time to annotate when a social relationship was first started or ended—“introduced_at” and “relationship_ended_on”. There is also another data property, “started_relationship_at_age”, that annotates an integer to mark the ego’s age when the relationship first occurred.

**Fig. 8:**
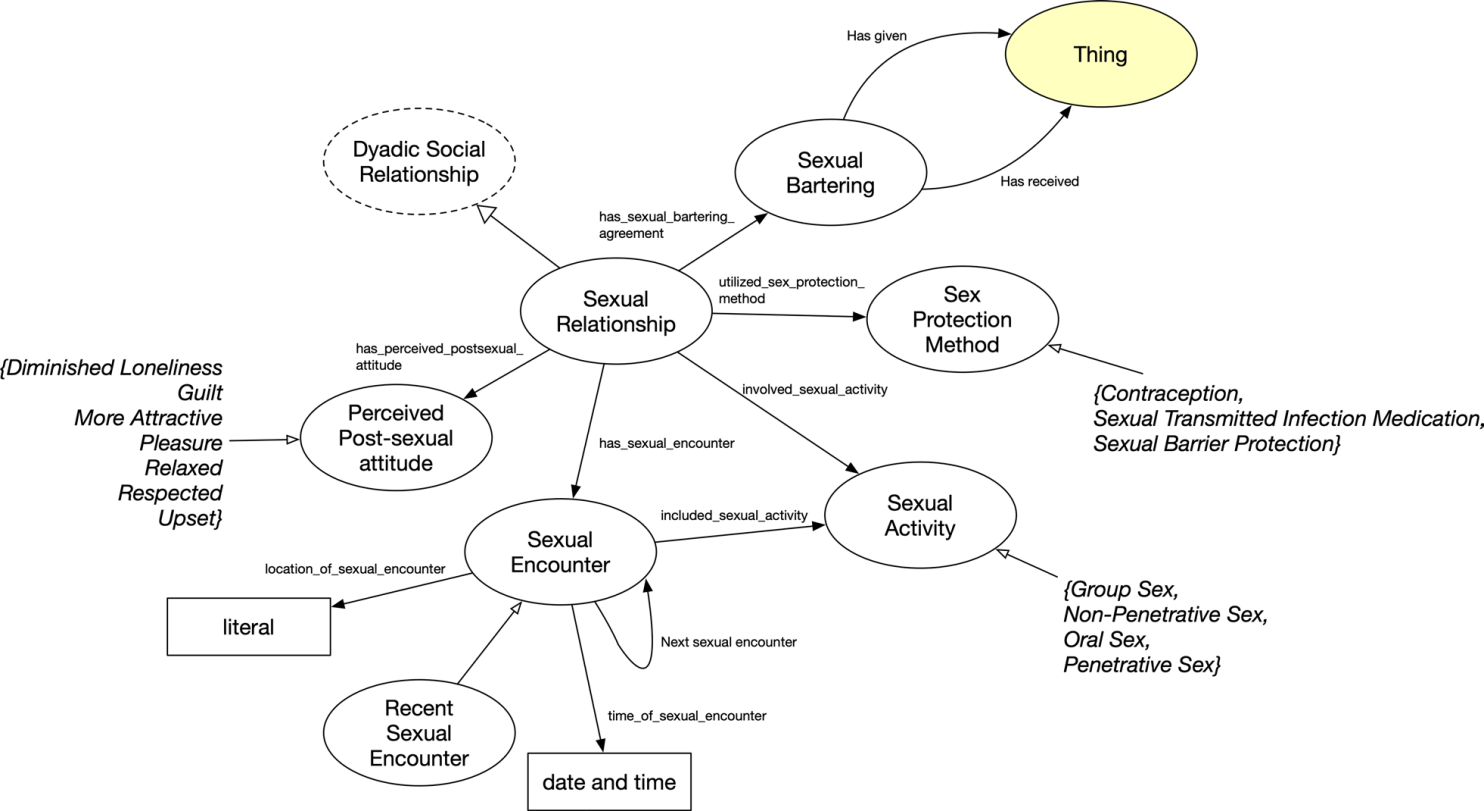
Sexual Relationship concept where classes are represented as circles and rectangles as data value types for data properties. Yellow highlights represents original FOAF ontology concept and property

Sexual Relationship, a subclass of Dyadic Social Relationship, inherits all the parent’s expressions, plus additional representations (Fig. 8). Sexual relationships may include bartering for sex and protection methods. The ADD Health survey also gauged participants’ perceptions of their sexual relationships (Perceived Post-Sexual Attitude). Sexual Encounters (i.e., all encounters with the alter) and Sexual Activity (i.e., type of activity) were represented under the Sexual Relationship concept. There are also data properties specifically for the Sexual Encounters class—“location_of_sexual_encounter” and “time_of_sexual_encounter”. The former annotates the setting of the encounter and the latter denotes the date and time of the event.

**Fig. 9:**
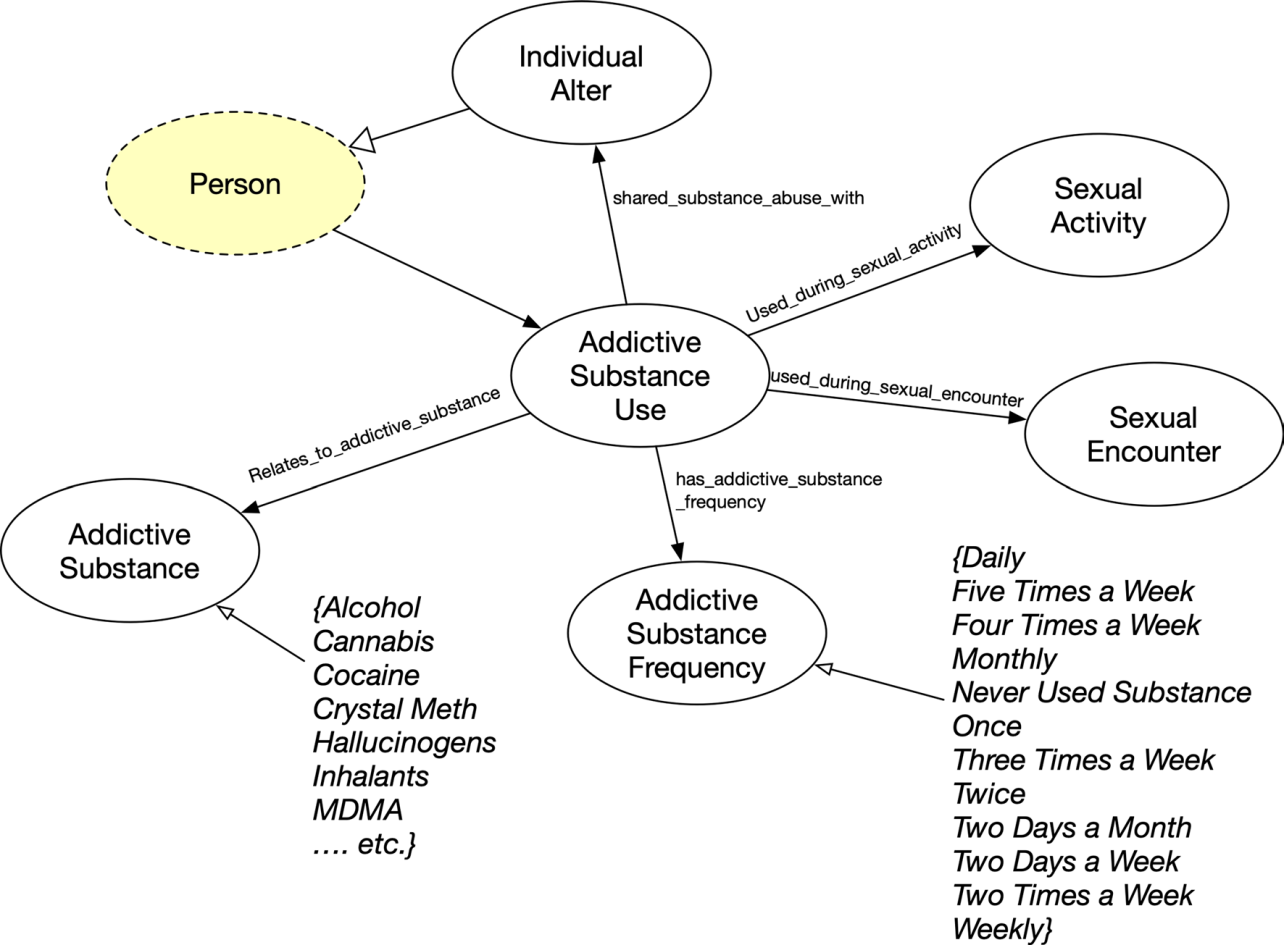
Addictive Substance Use concept where classes are represented as circles and rectangles as data value types for data properties. Yellow highlights represents original FOAF ontology concept and property

### Personal substance use

In Fig. 9, drug abuse information was represented in FOAF+ as Addictive Substance Use. Like the Dyadic Social Relationship concept, Addictive Substance Use may involve an individual’s partner or associate if the substance abuse activity was shared. The surveys also collected information on whether a substance was used during a sexual encounter or a sexual activity. Addictive Substance Use was an n-ary representation because of the complexity of the act, so the frequency (Addictive Substance Frequency) and the exact substance (Addictive Substance) were connected.

**Fig. 10:**
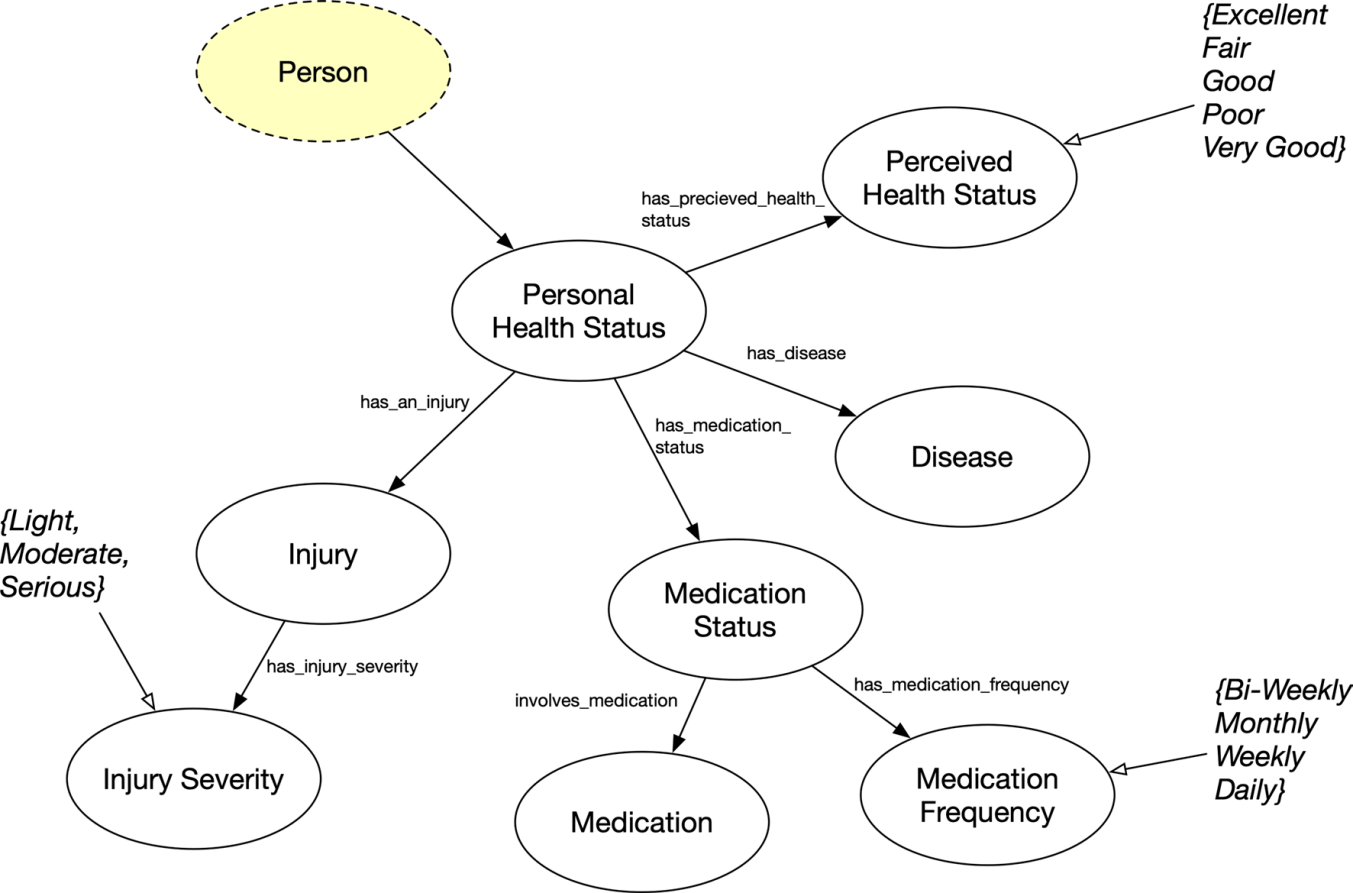
Personal Health Status concept where classes are represented as circles and rectangles as data value types for data properties. Yellow highlights represents original FOAF ontology concept and property

### Individual health status

While the surveys collected general health information, the Personal Health Status concept (Fig. 10) was not comprehensive, but it was sufficient for our purposes. Personal Health Status was also designed in an n-ary model, which included how people perceived their own health, what disease they might have, what medication they take and how often, and what injuries they have and the severity.

**Fig. 11:**
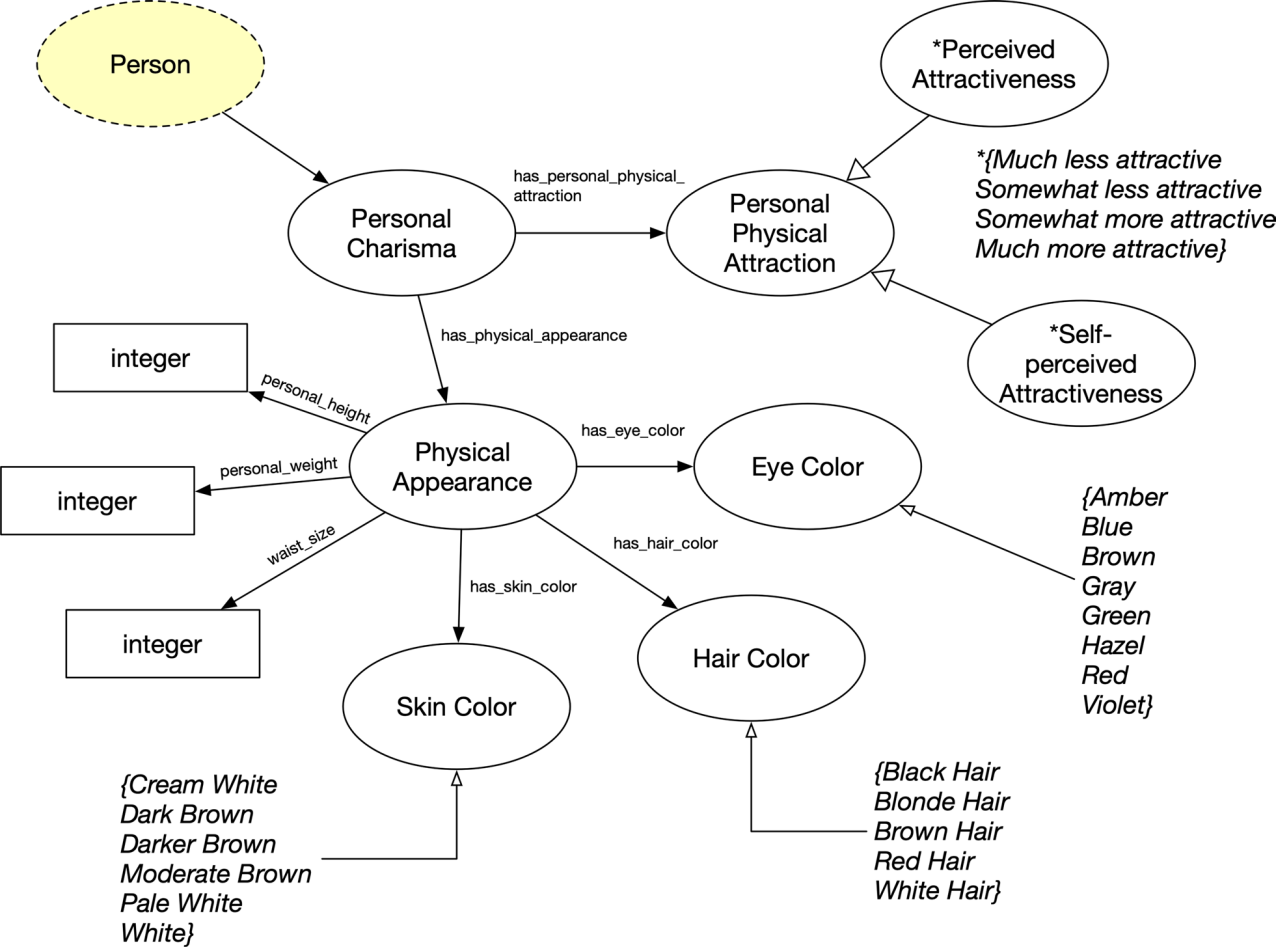
Personal Charisma concepts where classes are represented as circles and rectangles as data value types for data properties. Yellow highlights represents original FOAF ontology concept and property

### Social presence and self-perception

Finally, the Personal Charisma concept (Fig. 11) defines the overall presence of an individual. Both the YMAP and ADD Health Project surveys collected data relating to personal appearance and an individual’s perception about appearance. This was represented by Personal Physical Attraction and sub-concepts of Perceived Attractiveness and Self-Perceived Attractiveness. Also, basic physical characteristics (Physical Appearance class) were represented with Skin Color (Fitzpatrick scale [21]), Hair Color (Fischer–Saller scale [22]), Eye Color (Martin–Schultz scale), and other data property attributes like personal height, waist size, and weight as integer values.

## Results

Using the surveys, existing ontologies, and other research materials, like publications, we created the Friend of a Friend with Benefits (FOAF+) ontology, which enriched and extended the scope of the social network domain. FOAF+ has 713 classes, and 137 object properties, 130 data properties, and 312 instances. In comparison, the original FOAF contained 22 classes, 40 object properties, 27 data properties, and 0 instances.

**Table 1:** Evaluation scores comparing social network ontologies

	FOAF+ (minimal)	FOAF	VIVO (v.1.7)	$\mu$	$\sigma$	z-score
Syntactic	0.72	0.70	0.74	0.72	0.02	0.00
Lawfulness	0.97	0.94	1	0.97	0.03	0.00
Richness	0.49	0.46	0.49	0.48	0.02	0.58
Semantic	0.96	0.87	0.96	0.93	0.05	0.58
Interpretability	0.94	0.89	0.95	0.93	0.03	0.41
Consistency	1	0.92	0.99	0.97	0.04	0.69
Clarity	0.99	0.86	0.99	0.95	0.08	0.58
Pragmatic	0.43	0.05	0.47	0.32	0.23	0.49
Comprehensiveness	0.43	0.05	0.47	0.32	0.23	0.49
Overall	0.70	0.54	0.72	0.66	0.1	0.48

To attain an initial basic evaluation of FOAF+, we measured FOAF+ (minimal version) in comparison with other similar social network ontologies like VIVO (v.1.7) [23] and the original FOAF using semiotic metrics proposed by Burton-Jones and colleagues [24]. Table 1 presents the semiotic metrics produced by our automated tool (OntoKeeper—an iteration of our work [25, 26]) that implements the aforementioned metrics—*syntactic* (lawfulness, richness), *semantic* (interpretability, consistency, clarity), *pragmatic* (comprehensiveness). The *syntactic* metric produces a measure that describes the quality of the syntax through the diverse use of ontological features or axiom types (*richness*) and minimal syntactic violations (*lawfulness*). *Semantic* scoring is based on the quality of an ontology’s labels—nominal ambiguity (*clarity*), minimal inconsistency with labels (*consistency*), and if the term labels are meaningful (*interpretability*). *Pragmatic* score is the assessment of the ontology’s utility or usefulness. While there are three sub-scores for *pragmatic* scoring, we resorted to using one of them, namely *comprehensiveness* which measures domain coverage in comparison with similar ontologies. The other two—*relevancy* and *accuracy*—require external resources that are unavailable at the time (i.e. tailoring the scoring suite is one of the characteristics of this evaluation method [24]). For more details about the metrics, our previous papers [25, 27] and the Burton-Jones and colleagues’ paper [24] provide further discussion about the evaluation metrics.

In Table 1, we present the initial scoring for FOAF+ along with VIVO and FOAF’s. We computed the z-scores for each metric using the FOAF+ scores. With respect to the reported z-scores on Table 1, FOAF+ appear to have good *syntactic*, *semantic*, and *pragmatic* quality based on z-scores above 0 that indicates average to above average scoring. Essentially, for a first version of this ontology, FOAF+’s machine readability (*syntactic*, 0.72, z = 0.00) had average syntactic conformity (*lawfulness*, 0.97, z = 0.00) which is the percentage of axioms that are checked for conformity through OWL-API’s OWL2Profile interface, and utilizes a little more ontology features than average (*richness*, 0.49, z = 0.58) which is the total percentage of features used. FOAF+ has slightly better coverage based on the *pragmatic* score (0.43, z = 0.49) that represents the *comprehensiveness* rating - a measure of the size of the ontology. This size was based on the percentage total number of instances, classes, and properties when compared to the average of total of VIVO, FOAF, and FOAF+. FOAF+’s *semantic* score (0.96, z = 0.58) reveals decent term quality based on disambiguity of terms (*clarity*, 0.99, z = 0.58), comprehensible labels (*interpretability*, 0.94, z = 0.41)—which are scores based on number of word senses per term label. The semantic scoring also reveals less redundancy (*consistency*, 1, z = 0.69) that is derived from the number of term labels with no repetitive labels.

The overall quality score based on three branches was 0.70 (z = 0.48) which would indicate comparable quality alongside available social network ontologies. VIVO exceeded FOAF+ on many metrics which yielded a better overall quality score (0.72). It is assumed because of the maturity and the periodic maintenance of that ontology that VIVO would be of better quality than FOAF+ and FOAF. Nonetheless, for an initial comparison of FOAF+, FOAF+ is adequate as a prototype release based on its overall quality score.

### Use-cases towards generating social networks for public health analysis

#### Rules for deriving dyadic networks

Having elaborated the concepts, we tested the idea of an inferred dyadic social network based on knowledge encoded in FOAF+. Semantic Web Rules Language (SWRL) [28] was used to create some of the rules to conjure the network between individuals that could be potentially linked to survey data. The assumption was that with SWRL, we could show inferences of dyadic relationships for sexual connections between people as well as establish connections for disease transmission and sex work networks.

Before creating the rules we compiled a list of dyadic relationships to complement the “knows” relationship in FOAF. This included “sex with,” “transmitted STI,” and “procured sex from” (sex work). Also, “knows,” and “sex with,” relationships were defined as symmetrical links, “transmitted STI” was defined as an irreflexive link, and “procured sex from” was defined as an asymmetrical link.

When the reasoner that enacts the SWRL rules is executed, the inferred network will show sexual partnerships ($Person_{a}$ $\xrightarrow {sex\_with, knows}$ $Person_{b}$), sexual disease transmission ($Person_{a}$ $\xrightarrow {transmitted\_STI}$ $Person_{b}$), and sex work ($Person_{a}$ $\xrightarrow {procured\_sex\_from}$ $Person_{b}$). 
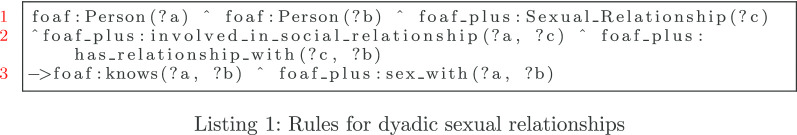


Listings 1 displays the SWRL rules that defined the dyadic connections. With Listing 1, in addition to their respective dyadic links, we wanted to encode their “knows” connection, as it is assumed that if one has sexual contact, they know the other party. 
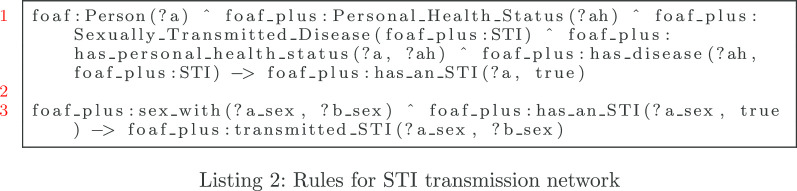


For the rule in Listing 2, we establish whether an individual has a sexually transmitted infection on line 1. On line 3, if the individual is infected and has sexual contact, the reasoner will generate an inferred connection of “transmitted STI” between the the infected individual to non-infected individual. 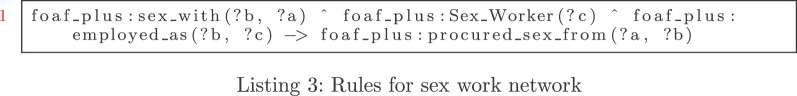


Listing 3 establishes the rules to generate a sex work dyad between individuals. Line 1 and line 3 are similar to dependent on whether one or the other individual was a sex worker. In line 1, if person “?a” was a sex worker then “?b” procured sex from “?a”. We also have an object property “offered sex to” that was an inverse of “procured sex from”.

### Inferred networks

After the rules were encoded, sample data were input to test each rule. Protégé’s built-in Pellet reasoner, which supports SWRL [29], was used to generate the dyadic connections for all encoded SWRL rules. We created a theoretical sample data to test if dyadic connections between individuals were generated for each rule.

#### Dyadic sexual relationship inference

**Fig. 12:**
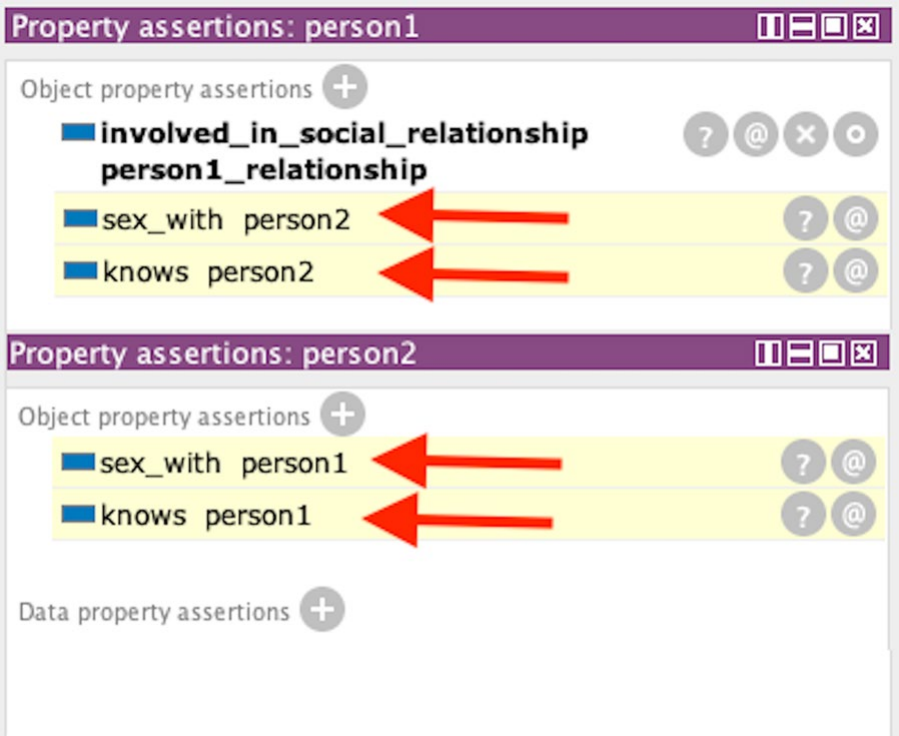
Sexual dyadic links between individuals, with links showing (inferred) “sex with” and “knows” with partners.Inference was produced using SWRL

For demonstrating the personal social relationship rule, we simply created two instances of a Person—“person1” and “person2”. We also created an instance of Sexual_Relationship—“person1_relationship”. For the “person1” instance we create an object property assertion with “person1_relationship” called “involved_in_social_relationship”. With “person1_relationship” we create an object property assertion of “has_relationship_with” with “person2” as the range. This instance of a n-ary relationship represents a dyadic relationship of a sexual nature where “person1” is the ego and “person2” is the alter. In essence, if a person has a sexual relationship with another, we presume they should know each other by default and that they had sexual contact with each other. With Fig. 12 shows the inferred relationship links between sexual contact and knowing one another. Both “person1” and “person2” had reciprocal (i.e., symmetrical) sexual contact and a knowing relationship.

#### STI transmission inference

**Fig. 13:**
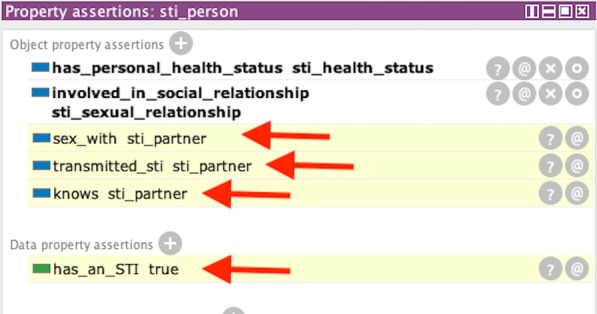
Sexual transmission knowledge is evoked from the rules in Listing 2. SWRL inference shows that individual (STI_person) has an sexually transmitted infection (“has an STI” = true) transmitted an STI (“transmitted STI”) to an individual (STI_partner). Inferences from Listing 1 are also shown with “sex with” and “knows”

Similar to previous example of dyadic inference, we define two Person instances—“sti_person” (ego) and “sti_partner” (alter)—along with an instance of Sexual_Relationship, “sti_sexual_relationship”. In addition we create an instance of Personal_Health_Status called “sti_health_status” that has an object property assertion of “has_disease” with “sti” (Syphilis) as the range. This health status instance data records having syphilis. We link the “sti_person” instance to this health status instance by instantiating an object property assertion of “has_health_status” with “sti_health_status” for the range. This indicates that the ego has a sexually transmitted infection. In summary, if a person (sti_person) who is the ego in a sexual relationship with his/her partner (“sti_partner”) has syphilis, we presume that not only they had sexual contact and know each other, but the ego establishes a presumptive transmission of a sexual transmitted disease. Figure 13 displays links that elicited the inferred information: sexual contact, disease transmission, knowing, and identifying the partner with an STI.

#### Sex work inference

**Fig. 14:**
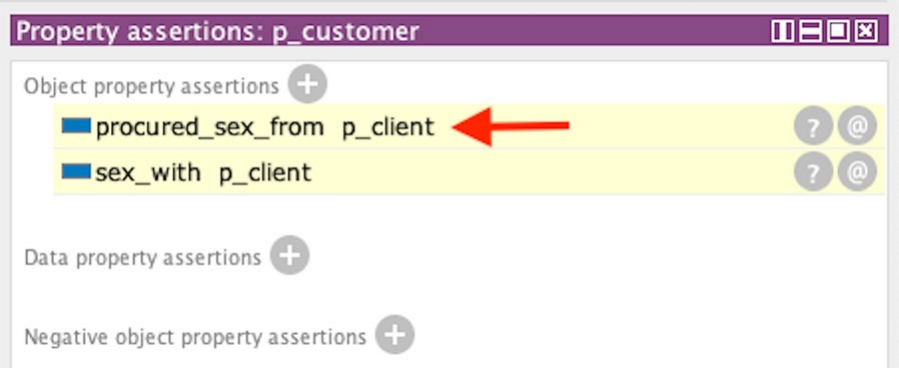
Inferred information for showing sexual contact with customer (p_customer) and trafficking link of “procured sex from” with sex worker (p_client) from Protégé. Inference was produced using SWRL

**Fig. 15:**
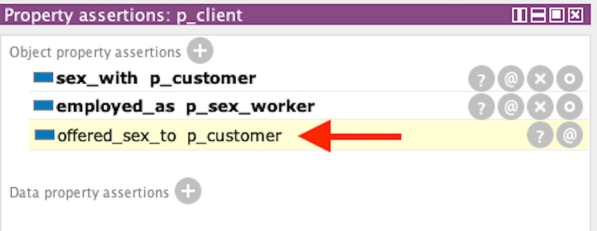
Inferred information for showing p_client offering sex to a customer (p_customer) Protégé. Inference was an inverse of the SWRL rule for Fig. 14

For the sex work rules, we create an instance of Person called “p_customer”, as an individual who solicits sex from a sex worker (“p_client”), who is also an instance of a Person. Since “p_client” is a sex worker we create an instance of Sex_Worker (Occupation) and create “p_client” object property assertion for “employed_as” linked to Sex_Worker as the range. We also establish that “p_client” had sex with “p_customer” by instantiating an object property assertion of “sex_with” with “p_customer” as the range. With knowledge that a sex worker had sex with a customer, we generalize that the sex worker offered it to the customer and that a customer had purchased the sex worker’s service. Figure 14 presents links that showed a customer (“p_customer”) purchasing sex from a client (“p_client”) and also a sexual contact. Complementing the sex work rule, Fig. 15 shows client(“p_client”) selling sex to customer(“p_customer”) with an inversed inference of procuring sex (i.e. “offer sex to”).

## Discussion

For each of the rules, we demonstrated the ontology’s reasoning capability to evoke dyadic networks between individuals for sexual contact, as well as generating inferred links for trafficking networks and disease transmission. For the latter, we did not account for any type of preventive measure like condoms or PrEP (Pre-Exposure Prophylaxis) use. Especially with YMSM (Young Men Who Have Sex With Men), inconsistent condom use or low PrEP uptake is not uncommon [30, 31]. While the ontology detailed extensive concepts for social interaction, we still must verify the encoded knowledge with domain experts, elaborate on other concepts like Disability and Community, and, lastly, add dyadic network connections beyond what was tested. Some of the concepts from FOAF+ like Medication or Disease served as proxy concepts that could be augmented in the future with well established ontologies.

The possible outcomes of this FOAF+ work is that it could help machines understand and interpret behavioral social data and to discover new relational links from survey data. Moreover, social network data is not immune to limited or missing tie information that can affect results [32, 33]. Through an ontology-driven approach we can help machines potentially discover missing link or tie information from social network data. Ontology-based query languages like SPARQL [34] and SQWRL [35] can enable querying the network data or produce network measures, like centrality [7]. Finally, this work may provide new analytical and surveillance methods for researchers.

### Future direction

Our focus is to expand the ontology’s domain scope and provide more elaboration of the conceptualization based on empirical data and review by social scientists. We intend to enlist domain experts in health social behavior to review the knowledge-base. This would involve the use of Hootation API that generates natural language statements from the ontology for expert revision (this software component is also integrated in OntoKeeper).

A future study goal will involve the use of survey data and using the final ontology to annotate that data. We also plan to complete YMAP (Young Men’s Affiliation Project of HIV Risk and Prevention Venue) survey data annotation in order to create an inferred network between participants. In the next stage, we aim to produce an actual network by linking to YMAP survey data with the ontology. This will include extensive preprocessing to identify potentially shared alters. The rules we introduced were general hypothetical ties that could be further elaborated in many areas of pubic health, but with the network data from YMAP we plan on developing specific SWRL rules that reflect the social structures of YMSM. By doing this, we can further reveal inferred information at an instance level within that data.

## Conclusion

Our project introduced an ontology-driven approach to logically inferring dyadic social networks between individuals. We enriched the knowledge structure of the FOAF ontology by means of surveys that describe social network relations between individuals. We produced the FOAF+ ontology, which included rules to define dyadic logical connections between people based on inferences from knowledge of the domain.

## Data Availability

Not applicable.

## Abbreviations

FOAF: Friend of a Friend
FOAF+: Friend of a Friend with Benefits
OGM: Ontology of general medicine sciences
OMRSE: Ontology of medically related social entities
SWRL: Semantic web rules language
ADD: Adolescent to adult project
YMAP: Young Men’s Affiliation Project of HIV Risk and Prevention Venue
PrEP: Pre-exposure prophylaxis
YMSM: Young men who have sex with men
